# Differential Viral-Host Immune Interactions Associated with Oseltamivir-Resistant H275Y and Wild-Type H1N1 A(pdm09) Influenza Virus Pathogenicity

**DOI:** 10.3390/v12080794

**Published:** 2020-07-24

**Authors:** Beatriz Vidaña, Pamela Martínez-Orellana, Jaime M. Martorell, Massimiliano Baratelli, Jorge Martínez, Lourdes Migura-Garcia, Lorena Córdoba, Mónica Pérez, Inmaculada Casas, Francisco Pozo, Lorenzo Fraile, Natàlia Majó, María Montoya

**Affiliations:** 1 Bristol Veterinary School, Faculty of Health Sciences, University of Bristol, Langford, Bristol BS40 5DU, UK; beatriz.vidana@bristol.ac.uk; 2 IRTA, Centre de Recerca en Sanitat Animal (CReSA, IRTA-UAB), Campus de la Universitat Autònoma de Barcelona (UAB), 08193 Bellaterra, Spain; pamela.martinez.phd@gmail.com (P.M.-O.); max2612@hotmail.it (M.B.); jorge.martinez@irta.cat (J.M.); lourdes.migura@irta.cat (L.M.-G.); lorena.cordoba@irta.cat (L.C.); monica.perez@irta.cat (M.P.); natalia.majo@uab.cat (N.M.); 3 Laboratory of Molecular Virology, International Centre for Genetic Engineering and Biotechnology (ICGEB), 34149 Trieste, Italy; 4 Departament de Medicina i Cirurgia Animals, Facultat de Veterinaria, Universitat Autònoma de Barcelona, 08193 Barcelona, Spain; JaumeMiquel.Martorell@uab.cat; 5 HIPRA Laboratories, Avda. la Selva 135, 17170 Amer (Girona), Spain; 6 Departament de Sanitat i d’Anatomia Animals, Universitat Autònoma de Barcelona (UAB), 08193 Bellaterra, Barcelona, Spain; 7 Centro Nacional de Microbiología, Instituto de Salud Carlos III (ISCIII), 28220 Majadahonda, Spain; icasas@isciii.es (I.C.); pacopozo@isciii.es (F.P.); 8 Department de Ciència Animal, Universitat de Lleida, 25198 Lleida, Spain; lorenzo.fraile@prodan.udl.cat; 9 Centro de Investigaciones Biológicas Margarita Salas (CIB-CSIC), Ramiro de Maeztu 9, 28040 Madrid, Spain

**Keywords:** influenza, immunopathology, pneumonia, pH1N1, resistance, oseltamivir

## Abstract

Oseltamivir is a common therapy against influenza A virus (IAV) infections. The acquisition of oseltamivir resistance (OR) mutations, such as H275Y, hampers viral fitness. However, OR H1N1 viruses have demonstrated the ability to spread throughout different populations. The objective of this work was to compare the fitness of two strains of OR (R6 and R7) containing the H275Y mutation, and a wild-type (F) pandemic influenza A (H1N1) 2009 (pdm09) virus both in vitro and in vivo in mice and to select one OR strain for a comparison with F in ferrets. R6 showed faster replication and pathogenicity than R7 in vitro and in mice. Subsequently, R6 was selected for the fitness comparison with the F strain in ferrets. Ferrets infected with the F virus showed more severe clinical signs, histopathological lung lesions, and viral quantification when compared to OR R6-infected animals. More importantly, differential viral kinetics correlated with differential pro-inflammatory host immune responses in the lungs of infected ferrets, where OR-infected animals developed a protective higher expression of type I IFN and Retinoid acid Inducible Gene I (RIG-I) genes early after infection, resulting in the development of milder disease. These results suggest the presence of early specific viral-host immune interactions relevant in the development of influenza-associated lung pathology.

## 1. Introduction

The pandemic A (H1N1) 2009 virus (referred to hereafter as pdm09) caused the first influenza A virus (IAV) pandemic of the 21th century. Currently, the pdm09 virus causes a predominantly mild disease, but it develops into severe lung pathology associated with mortality in some cases [1]. The pdm09 matrix protein (MP) gene came from the Eurasian swine lineage [2], which contains the S31N mutation conferring resistance to adamantine antiviral therapeutics [3]. Therefore, the pharmacological therapy against pdm09 infection depends on neuraminidase inhibitor (NAIs) antivirals, namely oseltamivir and zanamivir [3,4]. A single amino acid substitution at several positions in or around the NA active site is able to strengthen virus resistance against NAIs [5]. Among such NA substitutions, a histidine-to-tyrosine substitution at position 275 (H275Y) is one of the best-characterized and most commonly found oseltamivir-resistant (OR) mutations [6,7,8]. 

In general, it is considered that resistant mutations in the NA enzymatic position compromise viral fitness [9,10], as NAI-resistant viruses have been associated with deficient viral replication and spread in seasonal H1N1 and H3N2 strains [11,12]. However, seasonal H1N1 with the H275Y mutation emerged and expanded globally without oseltamivir selection [13]. Such an event demonstrated that viral fitness was no longer compromised by the resistance mutation. Several investigations have revealed the presence of diverse “permissive” mutations (R222Q, V234M, and possibly D354G) in seasonal and pdm09 viruses that allowed the addition of H275Y without compromising viral fitness [14,15,16]. 

Overall, OR pdm09 viral infections have generally been described in immunocompromised patients who have been subjected to antiviral treatment [17,18,19]. Regardless, community clusters of OR pdm09 viruses containing the H275Y mutation have been reported [20,21,22,23]. Despite the fact that the frequency of OR in pdm09 viruses has remained low (<2%) [24,25,26,27], there are indicators which suggest that pdm09 could permanently acquire the NA H275Y mutation, as happened with its predecessor, the seasonal H1N1 [10,23]. 

The aim of this study was to compare the virulence of two OR pdm09 strains (R6 and R7) and a wild-type (Wt) virus (F) in vitro and in mice, and to characterize differential innate viral-host immune parameters associated with viral pathogenicity in ferrets as a human model of disease. 

## 2. Materials and Methods

### 2.1. Oseltamivir-Resistant and Wild-Type Viruses

The three strains of human pdm09 viruses used in this study were isolated as previously described [28]. The A/Baleares/RR6121/2009 and A/Madrid/RR7495/2011 viruses bear the OR H275Y mutation and are hereafter referred to as R6 and R7, respectively. Both viruses were isolated from immunocompromised patients. The virus A/CastillaLaMancha/RR5911/2009, referred to here as F, was isolated from a fatal case of a patient without co-morbidities. Influenza viruses R6, R7, and F were grown in a Madin–Darby canine kidney (MDCK), as previously described [28]. R6 and R7 viral titers were 10^7.8^ 50% tissue culture infective dose (TCID_50_)/mL and 10^7.3^ TCID_50_/mL, respectively. The F virus had a titer of 10**^6.02^** TCID_50_/mL. Titers were determined using the Reed and Muench method [29]. Aminoacidic differences between viral strain-predicted proteins were identified. For each viral protein, the frequency of the amino acid at the mutated positions was calculated over an alignment greater than 1800 sequences of IAV recovered from the Global Initiative on Sharing Avian Influenza Data (GISAID) database.

### 2.2. In Vitro Infection and Viral Load 

MDCK cells were infected at 0.0001 multiplicity of infection (MOI) with OR pdm09 virus R6 and R7 and the Wt virus F. At 0, 6, 12, 24, and 48 h post-infection (hpi), supernatants were collected. Viral quantification was determined by TCID_50_ and a plaque assay determining PFU as previously described [28]. Virus infectivity in lung tissue samples from the mice virus was determined at 3, 5, and 7 dpi, as described by Almansa and colleagues [30]. 

In ferrets, viral secretion shedding in nasal swabs at 0, 1, 3, 6, and 10 dpi was determined by PFU for all available infected animals, as described before [28]. Nasal swabs were placed in 0.5 mL of DMEM (BioWhittaker^®^, Lonza, Verviers, Belgium) with 600 µg/mL penicillin and streptomycin, and frozen at −80 °C until further processing.

### 2.3. Ethical Statement

Experiments with mice and ferrets were performed following the animal use protocol approved by “Comissió d’Ètica en l’Experimentació Animal i Humana de la Universitat Autònoma de Barcelona” (Internal Register Number: 1124M2R) and the Ethical Animal Experimentation Commission of the Catalan Government (Register Number: 5767). Seven-week-old C57BL6/JOlaHsd (C57BL6) female mice (Harlan Laboratories, Barcelona, Spain) were housed in groups in experimental isolation cages at the biosafety level 3 (BSL3) facilities at the Centre de Recerca en Sanitat Animal (CReSA, Barcelona, Spain). Once mice were separated into different groups, they were kept for one week in acclimation. Animals were kept in standard housing cages and provided with commercial food pellets and tap water ad libitum throughout the experiment. Between eight and nine months of age, IAV seronegative ferrets (*Euroferret*, Denmark) were placed in BSL3 facilities at CReSA, in experimental isolation rooms, and then acclimated for 6 days. The animals were inhabited in standard housing cages for laboratory ferrets (F-SUITE Ferret Housing, Tecniplas, Italy) and were provided with commercial food pellets and tap water ad libitum throughout the experiment. 

#### 2.3.1. Mice Infection, Clinical Signs, and Sampling

C57BL6/JOlaHsd female mice were separated into three groups of 20 animals. Mice were assigned to the following three groups: Uninfected control group (C); R6-infected group (R6); and R7-infected group (R7). Each virus was inoculated separately by an intranasal route at a dose of 10^4^ PFU/mL. The control group remained uninfected. 

Mice were observed daily to record changes in body weight and clinical signs and any animal presenting severe weight loss of more than 20% compared to the weight at the start of the experiment, difficult labored breathing, or a prolonged inability to remain upright was humanely euthanized. Specimens of the serum and lung were acquired at 0, 3, 5, 7, and 14 dpi. In order to minimize suffering, blood samples were obtained under 5% isofluorane anesthesia. Euthanasia of mice was performed under anesthesia with 5% isoflurane with an intraperitoneal inoculation of penthobarbital. Subsequently, lungs were dissected by surgical methods. Lung tissues were weighed, homogenized, dry ice frozen, and stored at −80 °C until further use. 

#### 2.3.2. Ferret Infection, Clinical Signs, and Sampling

Sixteen ferrets were randomly assigned to three different experimental groups. The control group included three ferrets. The R6 and F group included eight ferrets each, intratracheally inoculated with 10^5^ TCID_50_/mL of the R6 or F pdm09 strain, respectively. Ferrets (*Euroferret*, Denmark) were neutered males between eight and nine months old and seronegative for IAV (influenza A antibody competition multi-species ELISA, ID Screen^®^, France). Intratracheal inoculation was performed under deep sedation of the animals with ketamine (5–10 mg/kg) (Imalgene 1000^®^ Merial, S.A., Spain) and medetomidine (0.05 mg/kg) (Domtor^®^ Pfiser, S.A., Spain), both administered subcutaneously, and after inoculation, animals were reverted from anesthesia with atipemazol (0.25 mg/kg), administered subcutaneously. Animals were monitored by a specialized veterinarian until they had fully recovered from sedation. 

Ferrets were monitored daily for clinical signs following a clinical score modified from [28]. Control animals’ clinical signs were monitored during acclimatization and at 0 dpi. 

Animals were euthanized by an intravenous injection of sodium pentobarbital (100 mg/kg) under anesthesia with ketamine (5–10 mg/kg/SC) (Imalgene 1000^®^ Merial, S.A., Spain) and medetomidine (0.05 mg/kg/SC) (Domtor^®^ Pfiser, S.A., Spain). Necropsies were performed on 1, 3, and 10 days post infection (dpi).

##### Ferret Acute Phase Proteins

Ferret serum APP haptoglobin (Hp) and serum amyloid A (SAA) were measured and quantified by an ELISA Haptoglobin Assay and Multispecies Serum Amyloid A Immunoassay, (Tridelta Development Ltd., County Kildare, Ireland). Sera samples were taken under sedation of the animals with butorphanol 0.5 mg/kg administrated subcutaneously, from the cranial vena cava at 0 dpi in control animals and all infected animals available at 0, 1, 3, 6, and 10 dpi. All samples were collected in 1 mL blood-heparine tubes. Heparine blood samples were centrifugated at 3000 rpm for 10 min at 4 °C to separate plasma samples. Plasma samples were store at −20 °C. Animals were individually monitored by a specialized veterinarian until they had completely recovered from sedation. Sampling at 0 dpi in all animals was performed while animals were anesthetized for viral inoculation, in order to minimize the handling and anesthetic procedures in the animals. 

##### Viral RNA Detection and IAV Antigen Expression and Quantification in Ferret Lungs

Viral RNA was extracted with the NucleoSpin^®^ RNA Virus Kit (Macherey-Nagel, Düren, Germany), following the manufacturer’s instructions, using a 5 mm square sample of the proximal mediastinic right caudal lobe of each ferret. The IAV M gene was quantified by TaqMan one-step quantitative real time RT-PCR (rRT-PCR) using primers, a probe, and the amplification conditions described in [31] using Fast7500 equipment (Applied Biosystems, Foster City, CA, USA). Individual samples were run in duplicate and each assay contained known positive and negative amplification controls that were also run in duplicate. Threshold cycle (Ct) values ≤40 were considered positive for IAV. 

The IAV antigen was also quantified in the lung by immunohistochemistry (IHC). Fixed paraffin-embedded tissues were stained with a primary antibody against the influenza A nucleoprotein and IAV antigen immune staining was quantified as previously described [32]. 

##### Ferret Innate Immune Gene Expression Profiles 

The gene expressions of IFNα, IFNβ, IFNγ, TNFα, IL-6, IL-8, CXCL10, and RIG-I were detected by two-step RT-PCR, as previously described [32]. 

#### 2.3.3. Histopathology

Mice and ferret lung sections were taken for histological examination, according to standard protocols, and stained with hematoxylin and eosin (HE) for examination under light microscopy. 

Cross sections of each mouse’s right lung lobes were separately evaluated, and semiquantitative assessments of IAV-associated microscopic lesions in the lungs were performed. The histopathological scoring grades assigned were ranked as follows: 0 (no lesions); 1 (mild to moderate necrotizing bronchiolitis); 2 (bronchointerstitial pneumonia characterized by necrotizing bronchiolitis and inflammation of close surrounding alveoli); and 3 (necrotizing bronchiolitis and diffuse inflammation of the alveolar parenchyma in the section). Microscopic lesion scores were assigned for each lobe, and the means were used for the final histopathological score for each animal.

#### 2.3.4. Hemagglutination Inhibition (HI) Assay

Antibodies against IAV were measured using an HI assay, as previously described [28]. Briefly, mice and ferret sera were treated overnight with four volumes of Receptor Destroying Enzyme (RDE) (Sigma-Aldrich SA, Madrid, Spain) solution (100 U/mL) at 37 °C. Then, five volumes of 1.5% sodium citrate were added to serum samples and incubated for 30 min at 56 °C. Treated sera were diluted 1:2 in PBS. Finally, sera were incubated for 1 h at 4 °C with 4 hemagglutination units of pdm09 and one volume of a 50% suspension of RBC. Controls of positive and negative sera were added to the assay. Positive results were considered when HI titers were >40.

### 2.4. Statistical Analyses

GraphPad Prism 6 (GraphPad Software, La Jolla, CA, USA) was used to perform data visualization. All statistical analysis was carried out using SPSS 15.0 software (SPSS Inc., Chicago, IL, USA). For all analyses, mice and ferrets were used as the experimental unit. The significance level (α) was established at 0.05. The Shapiro Wilk and Levene’s test were used to evaluate the normality of the distribution of the analysed quantitative variables and the homogeneity of variances, respectively. A normal distribution was not detected for any continuous variable. Therefore, a non-parametric test (Wilcoxon test) employing the U Mann–Whitney test to compare each pair of values was used to confront the different values collected for all of the parameters (weight loss, clinical signs, acute phase proteins, histopathology, viral load, and cytokine and viral sensing gene expression profiles) between groups (control, F, and R6 and R7 influenza virus) at all time points.

## 3. Results

### 3.1. Oseltamivir-Sensitive (F) and Oseltamivir-Resistant (R6) Replicate Faster than the R7 Virus In Vitro 

**Figure 1:**
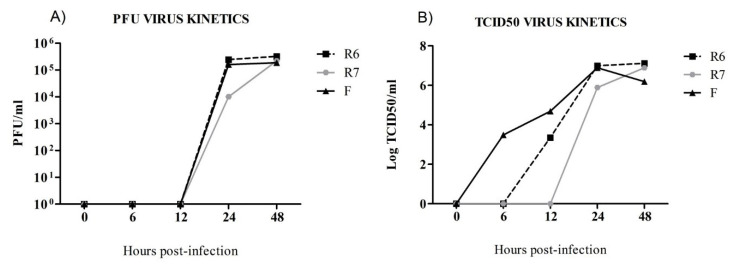
In vitro virus kinetics of pdm09 OR viruses (R6 and R7) were compared with a non-resistance pdm09 virus (F) and the viral titer was determined at 0, 6, 12, 24, and 48 h post-infection (hpi) by a plaque assay (**A**) and by 50% tissue culture infective dose (TCID_50_) (**B**).

Viral replication kinetics for R6, R7, and F viruses were examined by a regular virological method (PFU plaque assay and TCID_50_) and expressed in titers at different time points (Figure 1A,B). For PFU plaque assay results, viral titers from R6-infected cells (2.4 × 10^5^ PFU/mL) were similar to that for the F virus (1.6 × 10^5^ PFU/mL), but 24 times higher than the one from R7-infected cells (1 × 10^4^ PFU/mL) at 24 hpi (Figure 1A). No differences were observed at 48 hpi. When viral titers were measured by the TCID_50_ assay (Figure 1B), the progeny from F virus was detected as early as 6 hpi, whereas the one from the R6 virus was firstly detected at 12 hpi. At 24 hpi, higher viral titers were observed in F- (10^6.8^ TCID_50_/mL and R6- (10^6.9^ TCID_50_/mL) infected cells when compared with R7 (10^5.8^ TCID_50_/mL) (Figure 1B). Viral production was undetectable before 24 hpi in R7-infected cells. However, both OR viruses (R6 and R7) reached similar titers at 48 hpi, with values of 10^6.1^ and 10^6.8^ TCID_50_/mL, respectively. In conclusion, the R6 virus seems to replicate faster than the R7 virus in vitro.

### 3.2. R6-Infected Mice Presented Earlier Clinical Signs and Higher Mortality Rates than R7-Infected Mice

**Figure 2:**
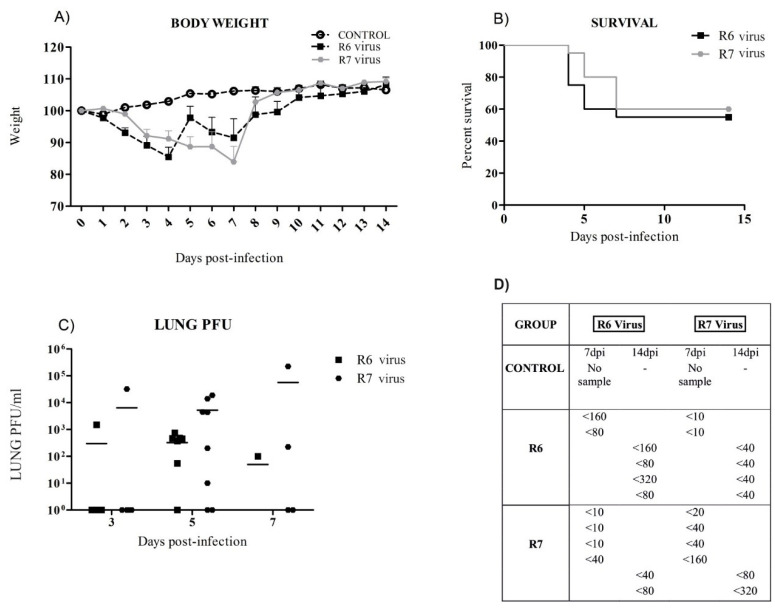
Influence on body weight (**A**), survival (**B**), (**C**) virus load in lungs, and (**D**) antibody response by haemagglutination inhibition (HI) in pdm09 oseltamivir resistance (OR)-infected mice. Groups of 20 mice were distributed as follows: Control group, R6-infected mice, and R7-infected mice. All values are the mean ± SEM of one experiment. (**B**) shows the percentage of survival of R6- and R7-infected groups. (**C**,**D**) exhibit individual values for each tested animal and the mean is represented by bars.

C57BL6 mice were intranasally infected with 10^4^ PFU of R6 or R7 viruses. The control group was not subjected to intranasal inoculation, remaining untreated. Animals were monitored daily for two weeks to control the body weight and survival (Figure 2A,B). Loss of body weight was not observed in untreated mice. The R6-infected group experienced a peak of weight loss at day 4 pi and R7-infected mice showed a similar peak but thereafter, at day 7 pi. During the first 2 dpi, the R6-infected group showed a statistically significant (*p* < 0.05) higher percentage of weight loss compared to R7-infected mice. Both infected groups experienced a statistically significant higher percentage of weight loss from days 3 to 7 pi compared to uninfected animals (*p* < 0.05). The statistical analysis of days 8 to 14 pi did not show any differences within groups; however, it is important to emphasize that body weight recovery was delayed in the R6-infected mice, whereas the R7 group started to exhibit an increased body weight at 7 dpi (Figure 2A). Importantly, 40% lethality was observed in the R6 group of animals at 4 dpi in comparison with 20% lethality for R7-infected mice. On the subsequent 2 dpi, lethality was maintained in R6-infected mice at 40% lethality and R7-infected mice at 30%. From day 7 to 14 pi, both infected groups exhibited 50% lethality (Figure 2B). All uninfected mice survived during the course of the study. Additionally, the percentage of survival was statistically significant (*p* < 0.0001) in control mice confronting the OR virus from day 4 pi until the end of the experimental infection course. 

### 3.3. Virus Replication in Mice 

Since lungs are primarily infected by IAV, samples of the target tissue from five infected mice were used to determine viral titers. At all time points, no statistically significant differences were observed within infected groups (Figure 2C). The viral burden increased gradually in the lungs from 3 to 7 dpi in R7 mice. Moreover, the lung viral load of R6-infected mice was reduced at 7 dpi and by 14 dpi, no infectious virus was detectable in any infected mice.

### 3.4. R6 and R7 Presented Similar Mice Antibody Responses 

The antibody response to the R6 and R7 virus was determined by the HI assay. Sera from days 7 and 14 pi were tested. Uninfected control mice were seronegative throughout the study period. At 7 dpi, the presence of hemagglutinin inhibiting antibodies was detected early in two mice from the R6 group and three from the R7 group. There was an HI antibody response observed in both infected groups against the virus used for infection in each group; however, few cross-reactive antibodies were confirmed at 14 dpi (Figure 2D). 

### 3.5. R6-Infected Mice Showed More Severe Histopathological Lesions than R7-Infected Mice 

**Figure 3:**
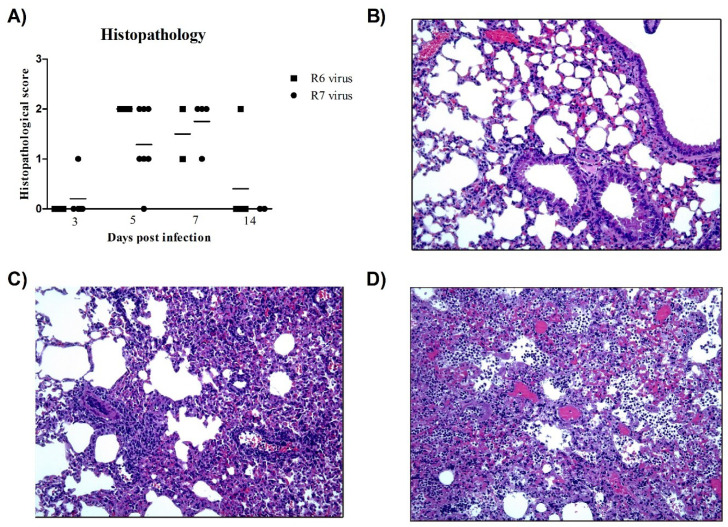
Histopathology of lung lesions in mice**.** (**A**) Histopathologic scoring of lung tissues examined. Individual values per animal with means represented by bars. (**B**) Control animals did not present any histopathological lesions at any time point. At 3 dpi, only one R7-infected mouse presented histopathological lesions consistent with bronchiolitis (**C**). At 5 dpi, all R6-infected mice presented with severe broncho-interstitial pneumonia (**D**). Hematoxylin and eosin (HE) stain (10× objective field).

Mice infected with the R6 virus presented higher histopathological scores than R7-infected mice (Figure 3A). Control animals did not present any histopathological lesions in the lungs at any time point (Figure 3B). At 3 dpi, only one R7-infected animal presented histopathological lesions consistent with mild necrotizing bronchiolitis, characterized by respiratory and glandular epithelial necrosis and mild lymphoplasmacytic infiltration (Figure 3C). At 5 dpi, all R6-infected animals presented moderate to severe bronchointerstitial pneumonia characterized by bronchi/bronchiolar and glandular necrosis with lymphoplasmacytic inflammation and neutrophilic and macrophagic infiltration in the adjacent alveoli (Figure 3D), whereas only three out of five R7-infected mice presented the same lesional score. At 7 dpi, three in four R7-infected mice and one in two R6-infeceted mice presented bronchointerstitial pneumonia. However, the low number of R6 mice examined at this time point may have lowered the histopathological score in comparison to the R7 group. At 14 dpi, one R6-infected mouse presented severe histopathological lesions consistent with bronchointerstitial pneumonia, while no R7-infected animals showed lesions at this time point. 

### 3.6. A/Baleares/RR6121/2009 (R6), (A/Madrid/RR7495/2011) (R7), and A/CastillaLaMancha/RR5911/2009 (F) Viruses Show Aminoacidic Differences

Viral sequences between F and R6 were compared and mutations PB2 225S, PB1 T20I, PB1 A652V, PA K716E, PA L589I, and PA S594G were found. Recognized mutations were screened in the GISAID database to detect the frequency in the pdm09 viruses (Table 1). Disparities in aminoacidic hallmarks were identified in the following proteins: PB2, PB1, PA, NP, HA, NA, and NS1. Predominant mutations fell into the viral polymerase complex proteins, especially into PB2 and PA. Notably, those in the F virus were mostly exceptional in the pdm09 viruses (minimum of less than 2.5%). The R6 virus, however, also showed some rare mutations with a frequency of 0.5%. The rest of the mutations were distributed among the rest of the proteins and many of them were unusual in the R6 virus. One mutation in the HA protein of the R6 virus is extremely rare (0.1%, just one sequence). Unfortunately, the domains into which they fall have been roughly identified and the functional implication of those mutations has not been previously recognized (apart from the oseltamivir resistance).

The R6 strain appeared more virulent than R7 and was selected for a comparison with the wild-type F strain in ferrets. Moreover, R7 was isolated two years later than R6 and F, which may have resulted in genetic changes within the HA and NA genes, making the Wt virus F more suitable for comparison with the OR virus R6. 

### 3.7. F-Infected Ferrets Presented More Severe Clinical Signs, and Higher Blood APP and Antibody Responses than the R6-Infected Group

**Figure 4:**
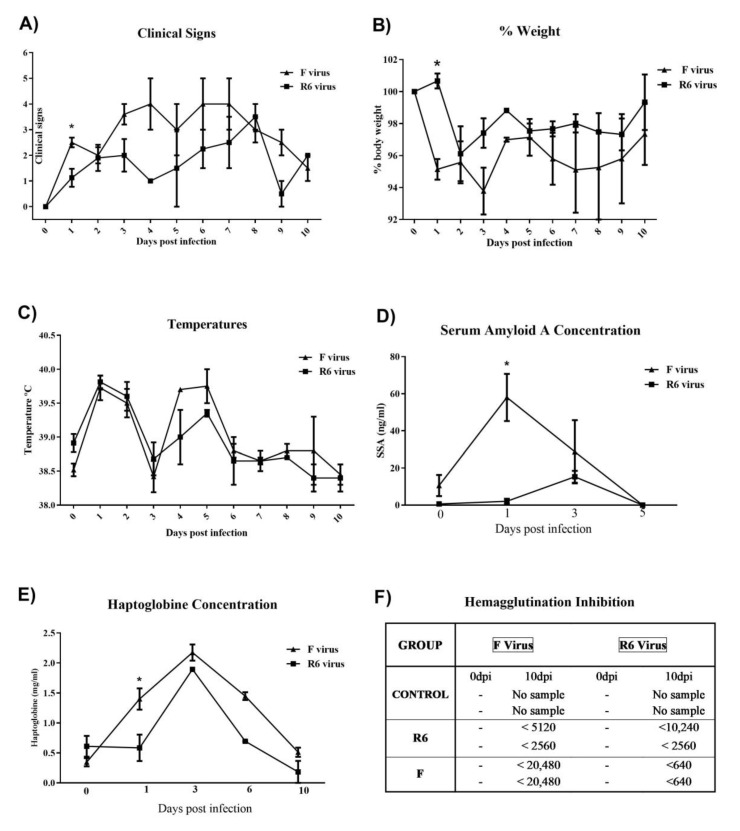
Clinical signs, APP’s levels, and antibody responses in ferrets. (**A**) Clinical score; (**B**) percentage of weight compared with weight at 0 dpi (100%); (**C**) body temperature; (**D**) SAA sera levels; (**E**) haptoglobin (Hp) sera levels. All values are represented by the mean and SEM ±. Statistically significant differences are represented by * (*p* value < 0.05). (**F**) Antibody response by HI.

Control animals did not present clinical signs, an elevation of APP blood levels, or IAV antibody responses. F-infected ferrets presented a higher percentage of weight loss and clinical scores than the R6 group (Figure 4A,B), and the weight loss percentage and clinical scores were higher in the F group than in OR-infected animals at 1 dpi (*p* < 0.05). At large, no differences in temperature variation between infected groups were observed (Figure 4C). Two APP, namely SAA and Hp, were analysed (Figure 4D,E). 

F-infected animals presented increased SAA levels from 1 to 3 dpi. F-infected ferrets showed higher SAA and Hp levels than R6-infected ferrets at 1 dpi (*p* < 0.05). Hp serum levels returned to basal levels at 6 dpi for animals infected with the R6 virus. However, the Hp levels of F virus-infected ferrets continued to increase until 10 dpi. F-infected ferrets showed higher antibody titers than the R6 group, determined by the haemagglutination inhibition (HI) assay in sera at 0 and 10 dpi (Figure 4F). 

### 3.8. F-Infected Ferrets Showed More Severe Histopathological Lesions and Higher IAV Antigen Immunostaining than R6-Infected Ferrets 

**Figure 5:**
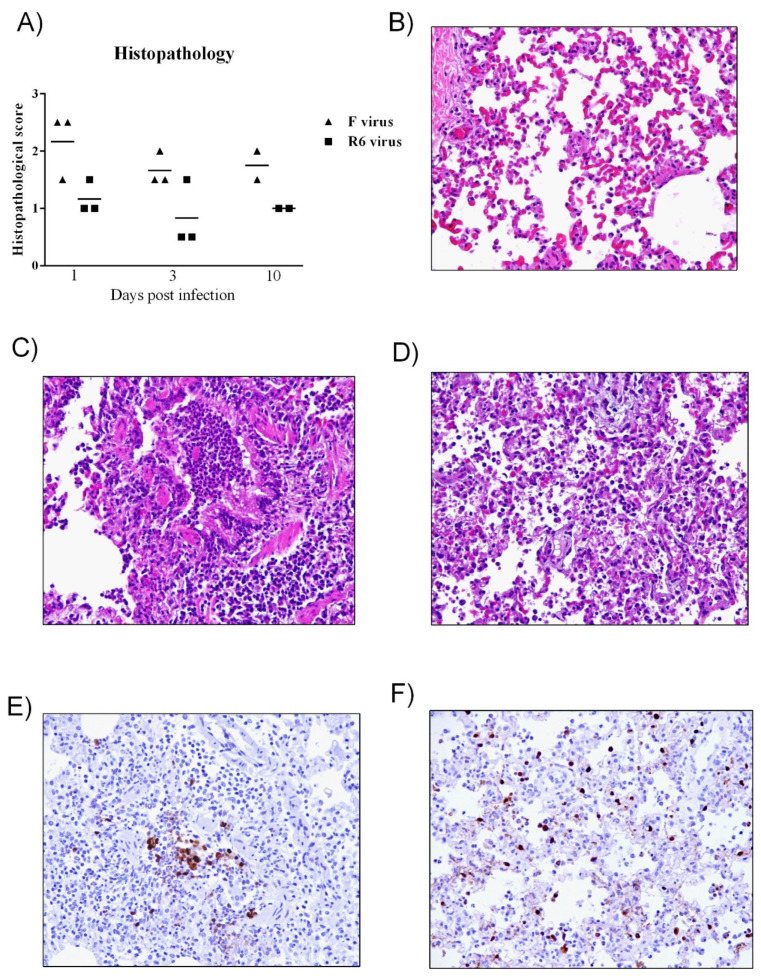
Histopathology of lung lesions and viral identification by immunohistochemistry (IHC) in ferrets**.** (**A**) Histopathological scores representing lung lesion severity. Individual values per animal with means represented by bars; (**B**) healthy lung in a control animal; (**C**) necrotizing bronchiolitis in one ferret infected with the R6 virus; and (**D**) diffuse alveolar damage in one animal infected with the F virus. (**E**) IAV-positive cells located in the bronchiolar epithelium in one animal infected with the R6 virus and (**F**) IAV-positive cells by IHC located in the alveolar septa in one ferret infected with the F virus. Images correspond to HE and immunohistochemical staining for the IAV antigen (20× objective field).

Gross lesions observed in infected animals were rare and subtle. At 1 dpi, one animal infected with the F virus displayed mild right lung consolidation, while no gross lesions were observed in R6-infected animals. At 3 dpi, two R6-infected animals displayed mild consolidation of caudal lung lobes and three ferrets infected with the F virus exhibited generalized interstitial emphysema or caudal lung consolidation. Animals infected with the F virus presented higher histopathological scores than R6-infected animals throughout the trial (Figure 5A). No gross or histopathological lesions were observed in the control group (Figure 5B). Histopathological lesions observed in the lungs of R6-infected ferrets were consistent with necrotizing bronchiolitis characterized by bronchiolar and glandular necrosis with lymphoplasmacytic inflammation (Figure 5C). F-infected ferrets exhibited bronchointerstitial pneumonia with variable grades of extension at 1 and 3 dpi. Diffuse alveolar damage characterized by high numbers of neutrophilic and macrophagic infiltrates within alveolar walls and alveolar spaces was also observed in the lungs of two animals infected with the F virus at 1 dpi (Figure 5D). Two R6-infected ferrets presented with mild bronchointerstitial pneumonia at 1 and 3 dpi. Control animal lungs were negative for IAV antigen expression by ICH. R6-infected ferrets mainly presented IAV antigen expression on bronchiolar epithelial and glandular cells in bronchiolar areas (Figure 5E), while F-infected ferrets presented viral antigen expression in bronchiolar and alveolar areas, with viral antigen expression observed in alveolar pneumocytes and macrophages (Figure 5F).

### 3.9. F-Infected Ferrets Presented Higher Viral Loads than R6-Infected Ferrets 

**Figure 6:**
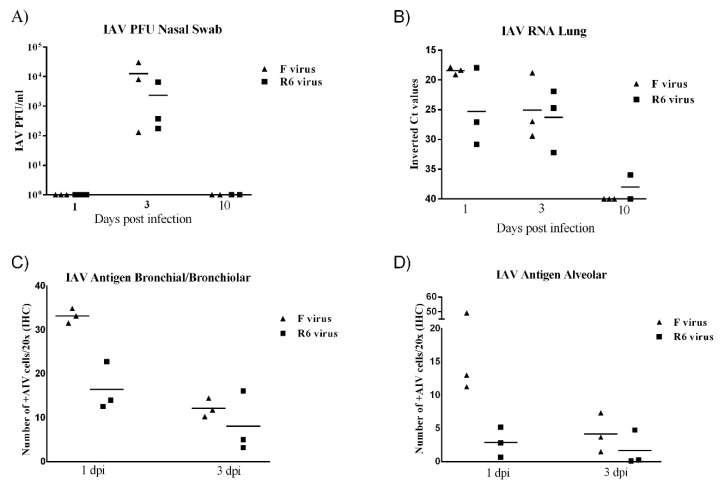
Viral quantification by PCR and IHC antigen cell accounts in ferrets. (**A**) Nasal swab viral shedding. Values represented as PFU/mL. (**B**) IAV rRT-PCR in the lungs. Values represented as inverted threshold cycle (Ct) values. (**C**) Immunohistochemical cell quantification of IAV-positive cells in bronchiol/bronchiolar areas. (**D**) Immunohistochemical cell quantification of IAV-positive cells in alveolar areas. Individual values per animal with means represented by bars.

Viral loads in nasal swabs and lung tissue samples were determined by a plaque assay and rRT-PCR, respectively (Figure 6). Control animals were negative for viral detection in nasal swabs and lung tissue samples. 

F-infected animals presented higher viral titers than R6-infected ferrets in nasal swabs at 3 dpi (Figure 6A). Viral RNA was detected in the lungs of both infected groups (F and R6) at 1 and 3 dpi, and in the lungs of one R6-infected ferret at 10 dpi. Viral RNA levels were higher in F-infected ferrets at 1 dpi, but not at 3 dpi, where RNA levels where similar (Figure 6B). Higher numbers of IAV immune-stained cells were detected at 1 dpi in comparison to 3 dpi in both infected groups. F-infected animals showed higher numbers of IAV-positive cells in alveolar and bronchiolar areas in comparison to R6-infected ferrets, particularly at 1 dpi (Figure 6C,D). 

### 3.10. F- and R6-Infected Animals Showed Different Innate Immune Genetic Profiles in the Lungs throughout Infection

**Figure 7:**
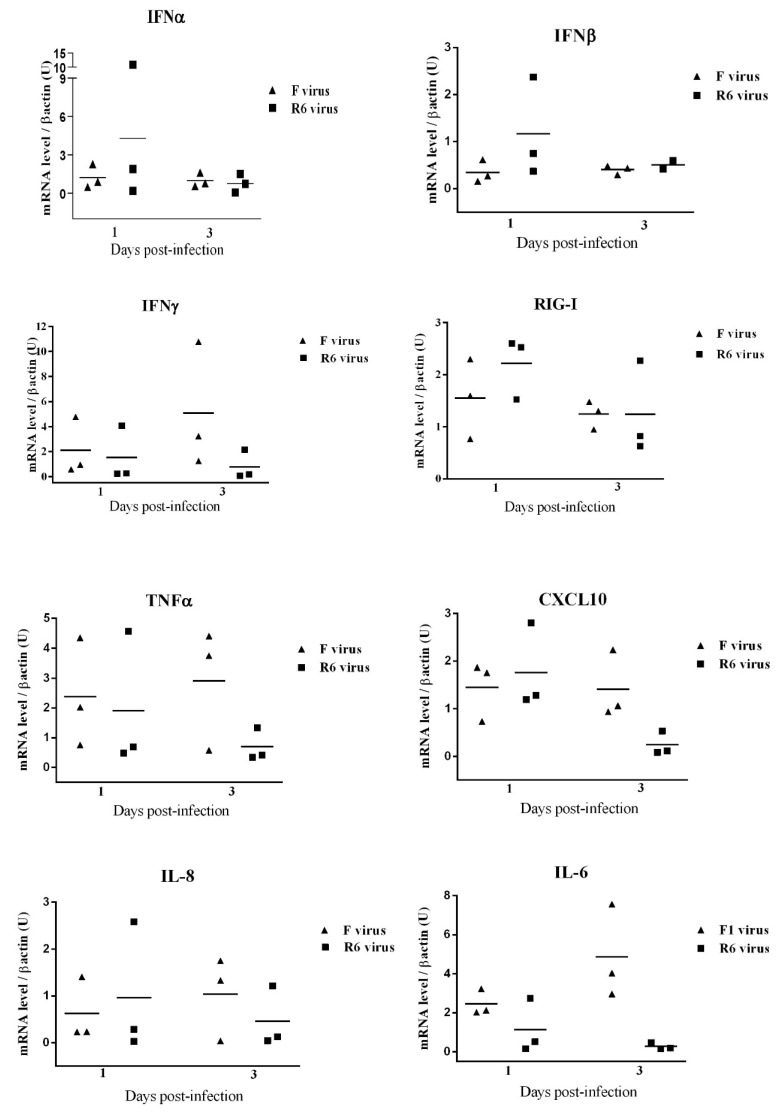
Cytokines and pattern-recognition receptor (PRR) quantification in the lungs of ferrets. Comparisons of the gene expression levels in lungs at 1 and 3 dpi by rRT-PCR. Gene expressions of IFNα, IFNβ, IFNγ and RIG-I, TNFα, CXCL10, IL-6, and IL-8. Individual values per animal with means represented by bars. No statistically significant differences were observed between groups.

Cytokines, chemokines, and the pattern-recognition receptor (PRR) already known to be related to IAV infection were quantified by rRT-PCR at 1 and 3 dpi from the lung tissue sampled. The F group exhibited a higher lung expression of the pro-inflammatory chemokine (C-X-C motif) CXCL10, IFNγ, and IL-8 at 3 dpi (Figure 7). In addition, higher mRNA levels of IL-6 and IFNγ were detected in the lungs of F-infected ferrets in comparison to the R6-group, at 1 and 3 dpi. In contrast, lungs from R6-infected ferrets exhibited a higher induction of the pattern recognition receptor Retinoid acid Inducible Gene I (RIG-I), type I IFNs (IFNα and IFNβ), and TNFα, at 1 dpi (Figure 7).

## 4. Discussion 

In general, OR pdm09 viral strains with the H275Y NA mutation are less virulent than Wt viruses [24,25]. However, several in vivo experiments have found conflicting results regarding their virulence in mice and ferret models [9,20,40,41,42]. This study investigated the viral pathogenicity of two OR pdm09 viral isolates containing the H275Y NA mutation in relation to a Wt pdm09 viral isolate and assessed the presence of distinctive viral-host immune interactions associated with viral pathogenicity in each case. 

The ability of the OR virus to generate infectious progeny in a host is still controversial. Despite some studies reporting a statistically significant impairment in viral growth in vitro [9], other groups’ analysis have not identified significant differences regarding the replicative capacity between the pdm09 Wt virus and its H275Y variant [43]. Here, different viral kinetics and virulence were observed between the OR viral strains, with R6 showing higher viral replication capabilities than the R7 isolate early after infection. In addition, in vitro assessment of the oseltamivir H275Y NA mutation seems to reduce viral fitness by means of viral replication when compared with the Wt F virus at the initial stages of infection, as previously described [15,44]. 

Subsequent to the in vitro study, a comparative study of virus growth fitness and pathogenesis was performed for R6- and R7-infected mice. The results revealed that both OR strains induced a fatal outcome, although with different degrees and kinetics. R6-mice experienced a 40% lethality and R7-mice a 20% lethality at 4 dpi. This result was consistent with the fact that R6 replicated faster in vitro. Importantly, 50% lethality in F virus-infected mice was only observed when a dose of 10^6^ PFU/mouse was used in a previous experiment performed by our collaborators [45]. Differences in viral kinetics were translated into differences in pathogenicity between OR viral strains. Mice belonging to the R6-infected group showed a statistically significant (*p* < 0.05) higher percentage of reduction in the body weight during the first 2 dpi compared to the R7 group of infection, which correlated with more severe lung histopathological findings of R6-infected mice. Curiously, higher lung viral loads were detected in R7-infected mice, which may suggest the presence of additional virus-host genetic mechanisms involved in pathogenicity other than enhanced viral kinetics. However, no statistical differences were observed within OR-infected groups. Previously, it was reported that the Wt pdm09 virus and H275Y mutant variant induced comparable mortality rates, weight loss, and lung titers in mice [15]. Our results are partially in agreement with this statement, but we observed dissimilar kinetics in mortality rates and loss of weight when the two OR viruses were compared. Surprisingly, R6-infected mice developed higher HA antibody titers than the R7 group, with a strong antibody response at 14 dpi in both infected groups, but the absence of cross-reactive antibodies. 

Amino acidic sequences of both viruses were compared, showing that F and R6 viruses exhibited differences in amino acidic signatures in several proteins, such as PB2, PB1, PA, NP, HA, NA, and NS1. It is worth mentioning that the majority of signatures of the F virus are rare in the pdm09 linage, and therefore, a possible correlation with F virus higher pathogenicity in comparison to R6 virus cannot be excluded. Nevertheless, none of the changes observed in the aminoacidic sequences have been characterized or described as playing a role in the domains in which they fall [46]. The large number of mutations found in the F virus polymerase complex indicates that it has been under a strong selection pressure, even greater than the surface glycoprotein and most probably related to the selected patient [45]. 

In relation to seasonal and pdm09 OR virus variants with the same NA mutation, it has been proposed that they might differ in replication or transmission fitness, depending on other viral components [47]. Previous studies have highlighted that a particular combination of residues in seasonal and pdm09 viruses, such as V234M or R222Q, which were not observed in earlier H1N1 viruses, resulted in an increased affinity of the NA cleavage balance. Moreover, those mutations were also significantly beneficial in compensating for the deleterious effect of the OR mutation [14,48]. Interestingly, it has been shown that viruses from both the Newcastle and Sapporo clusters contained permissive NA mutations V241I and N369K that enhance the replication and transmission fitness of viruses containing the H275Y mutation [49]. In addition, there is an increasing body of evidence that the NA275 mutation alone does not confer complete oseltamivir resistance in vivo, despite being repeatedly described in OR viral isolates, which may indicate that oseltamivir resistance mutations also exert effects in a genetic host-specific manner [50], in accordance with the results observed in this study. Therefore, the slower viral replication observed at early stages of infection in the R6 virus might be due to the lack of compensating mutations compared to the F group, but similar viral kinetics at later time points, further pointing to genetic host-specific interactions during early infection phases as being relevant in the pathogenicity of the virus beyond later viral replication capacities. 

More severe histopathologic lesions were apparent throughout the infection in ferrets belonging to the Wt pdm09 group, and more severe lesion scores were associated with increased in situ release of pro-inflammatory cytokines in the lungs and higher levels of acute phase proteins in the serum. In comparison with these results, F-infected animals exhibited significantly higher clinical scores compared to their OR counterparts. Several studies on ferrets and other mammals have already concluded that an elevated viral replication burden in the lungs and increased levels of APP are related to severe influenza virus infection [28,51,52].

The lung immune response in ferrets presented high individual variability within groups and no statistically significant results were observed between infection groups. However, F-infected ferrets showed a pattern characterized by a higher induction of pro-inflammatory cytokines IL-6, IL-8, CXCL10, TNFα, and IFNγ, particularly at 3 dpi. It has previously been described that the presence of a viral antigen in alveolar areas, together with the up-regulation of pro-inflammatory cytokines, is a marker of severity during pdm09 infection, both in humans and animal models [32,53,54]. Therefore, it is conceivable to suggest that lung lesions and clinical signs observed in F-infected animals might be, in part, a consequence of the lung’s excessive pro-inflammatory response as a result of the spread of the virus to alveolar areas. 

These results support previous studies which have hypothesized that OR-hampered virus growth in vitro may be a result of a reduced NA enzyme efficiency, which delays the release of progeny virions from the host cell surface. This delay would probably not affect the final virus exert, but would allow the host’s first-line innate immune defense to neutralize the virus in in vivo experiments [9]. Several studies have indicated that the correct and specific regulation of the innate immune response dynamics, particularly the up-regulation of IFN type I genes, are protective factors in the pathological outcome after pdm09 infection [32,55,56,57]. In agreement, our study showed that R6-infected ferrets presented a higher expression of interferon I and RIG-I genes, which was correlated with a lower viral load at 1 dpi. These observations could indicate that the host innate immune response better identified the R6 virus due to the slower viral growth at the early stages. In this context, such slower viral growth would allow the host to limit viral infection to bronchiolar areas, as shown by IAN antigen immunostaining in the lungs, avoiding the release of detrimental pro-inflammatory cytokines and resulting alveolar damage.

Overall, the present study provides further evidence about the important role of an efficient early innate immune response in the outcome of disease after influenza virus infection. Therefore, understanding the interaction among both viral genetic features inside the host immune genetic context is required for the development of future therapies against IAV infection.

## Figures and Tables

**Table 1:** Aminoacidic sequence comparison of the F and R6 strains. Aminoacidic differences between the two strains. Mutation frequency and the domain into which they fall were also annotated.

Protein	Portion Compared	Mutated Position	F	R6	Frequency	Domain	Ref.
**PB2**	1-757	127	Y	H	127H 98.8% (1837)	N-terminal-PB1,NP interaction	[33]
		191	K	E	191E 97.5% (1812)		
		221	T	A	221A 99.7% (1854)		
		660	K	R	660R 2.5% (47)	627Domain – host range	[34]
**PB1**	1-757	257	T	A	257A 0.5% (10)	Nucleotide binding site	[35]
**PB1-F2**	1-11 TRUNCATED	-					
**PA**	1-716	328	R	K	328K 99.8% (1855)	PB1 interaction	
		529	N	D	529D 99.8% (1855)		[36]
		716	K	E	716E 0.5% (9)		
**NP**	2-497	400	R	K	400K 98.8% (1836)	Body	[37]
**HA**	34-349	38	E	K	38K 0.1% (1)	Fusion HA1 chain	[38]
		127	L	S	127S 99.8% (1860)	Receptor binding HA1 chain	
**NA**	188-420	275	H	Y	275Y 0.8% (14)	Head domain	[39]
**M1**	1-252	-					
**M2**	1-97	-					
**NS1**	1-219	93	M	I	93I 2.2% (40)	N/A	
**NS2**	1-121	-					
